# The Role of Oligomerization and Cooperative Regulation in Protein Function: The Case of Tryptophan Synthase

**DOI:** 10.1371/journal.pcbi.1000994

**Published:** 2010-11-11

**Authors:** M. Qaiser Fatmi, Chia-en A. Chang

**Affiliations:** Department of Chemistry, University of California, Riverside, Riverside, California, United States of America; University of Parma, Italy

## Abstract

The oligomerization/co-localization of protein complexes and their cooperative regulation in protein function is a key feature in many biological systems. The synergistic regulation in different subunits often enhances the functional properties of the multi-enzyme complex. The present study used molecular dynamics and Brownian dynamics simulations to study the effects of allostery, oligomerization and intermediate channeling on enhancing the protein function of tryptophan synthase (TRPS). TRPS uses a set of α/β–dimeric units to catalyze the last two steps of L-tryptophan biosynthesis, and the rate is remarkably slower in the isolated monomers. Our work shows that without their binding partner, the isolated monomers are stable and more rigid. The substrates can form fairly stable interactions with the protein in both forms when the protein reaches the final ligand–bound conformations. Our simulations also revealed that the α/β–dimeric unit stabilizes the substrate–protein conformation in the ligand binding process, which lowers the conformation transition barrier and helps the protein conformations shift from an open/inactive form to a closed/active form. Brownian dynamics simulations with a coarse-grained model illustrate how protein conformations affect substrate channeling. The results highlight the complex roles of protein oligomerization and the fine balance between rigidity and dynamics in protein function.

## Author Summary

Conformational changes of enzymes are often related to regulating and creating an optimal environment for efficient chemistry. An increasing number of evidences also indicate that oligomerization/co-localization of proteins contributes to the efficiency of metabolic pathways. Although static structures have been available for many multi-enzyme complexes, their efficiency is also governed by the synergistic regulation between the multi-units. Our study applies molecular dynamics and Brownian dynamics simulations to the model system, the tryptophan synthase complex. The multi-enzyme complex is a bienzyme nanomachine and its catalytic activity is intimately related to the allosteric signaling and the metabolite transfer between its α– and β–subunits connected by a 25-Å long channel. Our studies suggest that the binding partner is crucial for the ligand binding processes. Although the isolated monomers are stable in the ligand–free state and can form stable interaction if the substrate is in the final bound conformation, it has higher energy barrier when ligand binds to the active site. We also show that the channel does not always exist, but it may be blocked before the enzyme reaches its final bound conformation. The results highlight the importance of forming protein complexes and the cooperative changes during different states.

## Introduction

The formation of protein oligomeric units often produces increased stability with improved function for the multi-enzyme complexes [1]. The co-localization of protein subunits can shape the active sites, allow allosteric cooperativity, provide an additional level of signaling or regulation, and even permit channeling of intermediates during an enzymatic turnover, which are some of the prime concerns in protein chemistry from the mechanistic point of view [2]–[10]. Such protein dynamics are long recognized to be intimately linked to enzymatic catalysis, but their relationship is exceedingly challenging to delineate [11]. Several experimental and computational studies have probed these fundamental enzymatic processes and their relationships and have provided invaluable insights into the molecular mechanisms [12]–[17]. Hemoglobin is one of the classical and well-studied proteins that exhibit large-scale ligand-induced conformational changes and allosteric cooperativity during the regulation of oxygen transportation. However, for a more complicated and larger system such as tryptophan synthase (TRPS), understanding protein function in relation to the protein dynamics and formation of the multi-enzyme complex becomes even more challenging.

**Figure 1:**
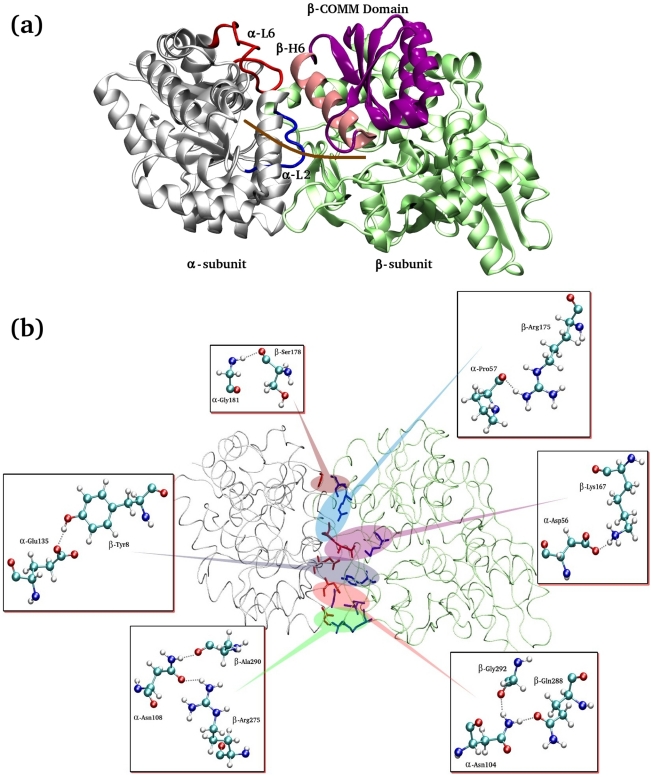
Structure of the α/β–dimeric unit of tryptophan synthase. (a) A labeled diagram of the α/β–dimeric unit of tryptophan synthase (TRPS). The important regions have been color coded: blue for α–L2 (residues α53–60), red for α–L6 (residues α179–193), purple+pink for the β–COMM domain (residues β102–189), and pink for the β–H6 of COMM domain (residues β165–181). The approximate location of the interconnecting channel is shown as a solid brown line. (b) The network of H–bonds at the α/β–interface of the TRPS dimeric unit. Some of the H–bonds play key roles in allosteric communications and the substrate channeling process. The interacting pair of residues is zoomed in, and the formation of possible H–bonds is shown in small panels.

The current work investigated TRPS, a pyridoxal 5′-phosphate (PLP)-dependent αββα protein complex that catalyzes the last 2 steps of tryptophan biosynthesis in bacteria, fungi and plants. Research studies conducted over the past 40 years have revealed interesting structural, dynamic and mechanistic features of this protein. The protein was the first known enzyme to exhibit 2 distinct catalytic activities modulated by allosteric and synergistic interactions and demonstrating an intermolecular substrate channeling process through a 25-Å long tunnel without exposing the intermediate to the environment (see Figure 1(a)). The α–subunit of TRPS resembles TIM barrel protein and is composed of 2 functionally important α–loops, L2 (α–residues 53–60) and L6 (α–residues 179–193), that surround the α–active site. The significant contributions of these loops in the α–catalysis and α/β–intersubunit communications have been widely recognized by both experimental and computational work [18]–[23]. Within the superfamily of PLP-dependent enzymes, the β–subunit of TRPS is classified as fold type II (see definition in Text S1) [24] and contains a movable communication domain (COMM domain; β–residues 102–189). The β–H6 of the COMM domain (residues 165–181) preferentially interacts with flexible α–L2 and α–L6 and mediates intersubunit allosteric communication. Both α– and β–subunits can adopt open and closed conformations. A fully closed conformation is proposed to be the active state of the protein in terms of catalysis and substrate channeling.

Although the isolated α– and β–monomeric units of TRPS can independently catalyze the α– and β–reactions, respectively, the rate is very slow [25]–[27]. Steady–state kinetic studies [28] revealed that the rate of the α–reaction in the isolated α–subunit is ∼100 times slower than that in the αββα tetramer of *Escherichia coli* TRPS, which has 84% identities and 94% similarities with the *Salmonella typhimurium* TRPS used in our simulation studies. This observation reflects a strong synergistic effect of subunits on the α–catalysis in the multi-enzyme complex. However, the synergistic effects on the β–catalysis are less pronounced. The rate of the β–reaction in the isolated β–subunit of *Zea mays* TRPS (ZmTSB1), which shares 96% identity with the bacterial β–subunit of TRPS, is only 1.5 times slower than the oligomeric TRPS of *Z. mays* [29].

While studying the stability of TRPS, Yutani and co–workers found that the isolated α– and β–subunits of *Pyrococcus furiosus* TRPS, which share 35% and 59% sequence identity with the α– and β–subunits of the *S. typhimurium* TRPS, respectively, are highly stable [30]–[31]. The study concluded that entropic effects are the major factors contributing to the stability. Similar results have been observed for *Thermus thermophilus*, a hyperthermophile with 30% and 55% identical amino acid sequences to the corresponding α– and β–subunits of the *S. typhimurium* TRPS, which indicate the importance of entropic effects in stability of the monomeric subunits [32]. Other kinetic studies investigated the homologs of the *S. typhimurium* α–subunit, such as BX1 (33% identical to the *S. typhimurium* α–subunit) and indole-3-glycerol phosphate lyase (IGL) from *Z. mays*. Both enzymes can efficiently catalyze the α–reaction without the other protein partner, but BX1 and IGL are about 1400 and 1150 times, respectively, more efficient than the isolated α–subunit of the *E. coli* TRPS [33]. The faster reaction rate for BX1 may be due to a highly stable Glu134 (structurally and functionally equivalent to the α–Glu49 of TRPS). Unlike the flexible α–Glu49 of TRPS, Glu134 of BX1 is rigid and preferably stays in the active conformation [34]. This finding suggests that efficient catalysis may require a fine balance between stability and flexibility of enzymes, although the detailed molecular aspects of such linkages are not clear.

In this study, we addressed fundamental questions of protein chemistry, including 1) the importance of oligomerization of protein subunits, 2) understanding subunit cooperativity and correlative motions, 3) the linkage between allostery and cooperativity with protein function, and 4) protein conformational changes in substrate channeling. We performed several sets of explicit water molecular dynamics (MD) simulations of α/β–dimeric and isolated α– and β–monomeric units of the *S. typhimurium* TRPS with and without ligands. Notably, the isolated α– and β–monomeric units are folded proteins and are stable in solution experimentally, but their catalysis rates are reduced [34]. The trajectories were analyzed, and intra– and inter–subunit correlated motions were illustrated. The ligand–protein interaction energies, entropic effects, and H–bond networks were also studied. Brownian dynamics simulations with a coarse-grained model were performed on selected protein conformations from the MD simulations to study substrate channeling.

## Materials and Methods

### Construction of the ligand–free (LF) open conformation α/β–dimeric unit

Since the protein data bank only contains α–subunit with a closed α–L6 loop, we performed a 15-ns MD simulation with a generalized Born (GB) implicit solvent model to obtain an open α–L6 loop conformation [35]. This method has been already employed for studying the HIV-1 protease flaps to successfully demonstrate the open and closed states of this protein [36]. The initial structural coordinates for the α–subunit were obtained from the *Salmonella typhimurium* TRPS (PDB entry 2J9X); the α–site ligand was manually removed [37]. The coordinates of three missing residues (Ala190, Leu191, and Pro192) in the α–L6 loop were taken from a completely closed *S. typhimurium* TRPS (PDB entry 3CEP) [38]. After a subsequent minimization, equilibration and MD simulations with the GB model in the Amber package [39], several open conformations of the ligand–free α–subunit were collected on the basis of the distance between α–Thr183 (α–L6) and α–Asp60 (α–L2). The open conformations of the ligand–free α–subunit were combined with a ligand–free open β–subunit (PDB entry 1QOQ) to construct several ligand–free TRPS with open α– and open β–subunit [40]. The modeled α/β–dimeric units were minimized and equilibrated in explicit water. The systems were then subjected to a minimum of 13–18 ns of explicit MD simulations and important distances were subsequently analyzed. The most stable ligand–free α/β–dimeric unit in terms of smooth distance fluctuations was then selected for a 60-ns MD simulation by use of the NAMD 2.6 program [41].

### Construction of the ligand–bound (open conformation) and ligand–bound–reference (closed conformation) dimeric units

A ligand–bound complex was constructed by placing both α– and β–site ligands in the binding sites. IGP was docked into the α–site of the ligand–free complex obtained from the procedure described in the previous section (the detail parameters of protein–ligand docking are given in Text S1). Since the side-chains of the α–site produced considerable changes during the free protein simulation (in particular the α–Phe212), molecular docking programs could not reproduce the crystal structure conformation of IGP. Therefore, the substrate was manually placed into the binding site, and the distances of catalytically important residues α–Asp60 and α–Glu49 with IGP were maintained, as suggested by experiments. The β–site ligand, aminoacrylate, was docked into the β–subunit of the ligand–free α/β–complex by use of the Autodock4 package [42]. The choice of IGP and aminoacrylate as ligands for α– and β–sites, respectively, ensures the closed conformation of the α/β–complex. The system containing α– and β–site ligands is termed the ligand–bound (LB) complex. After subsequent minimization and equilibration, a 100-ns MD trajectory was collected to observe the possible ligand-induced conformational changes in the complex. Since the simulation may require a very long time (probably a couple hundred ns long) to exhibit the switching of the α– and β–subunits from open/semi-open (LB state) to the completely closed states, we also run a reference simulation with a completely closed conformation. Therefore, another TRPS system, with IGP and aminoacrylate in the α– and β–site, respectively, was prepared by using the initial coordinates from a crystal structure (PDB entry 3CEP). This is our reference structure with completely closed α– and β–subunits, which we termed the ligand–bound–reference (LBR) complex. We created a 50-ns MD simulation after subsequent minimization and equilibration processes.

### Construction of the isolated monomeric units of TRPS

The isolated monomeric units for all three states (LF, LB and LBR) were simply prepared by splitting the α/β–dimeric units into their subsequent α– and β–monomers, so that the initial geometries of isolated monomeric units were exactly the same as their corresponding subunits in the dimeric unit for comparison.

### Computer simulation protocol

For the molecular dynamics simulations, the ff03 amber force field and general amber force field (GAFF) were applied to both α/β–dimeric and isolated α– and β–monomeric units (LF, LB and LBR TRPS complexes) [43]–[44]. An antechamber was used to create the topology and coordinate files for the ligands [45]. The protonation states for histidines, aspartates and glutamates were assigned by the MCCE program [46]. The TRPS dimeric units contain one α– and one β–subunit, whereas isolated monomers contain only one of each subunit.

Although no substrates bound to the LF dimeric and isolated monomeric units, a PLP molecule was kept as a co-factor in the β–active site. The systems were electronically neutralized by the addition of 14 Na^+^ ions for the α/β–dimeric units and 6 and 8 Na^+^ ions for the isolated α– and β–monomeric units. The LB TRPS represents a transition stage of the ligand binding process and was constructed by manually docking a substrate into a free subunit (see reference [47] for details). The system includes 3-indole-D-glycerol-3′-phosphate (IGP) in the α–active site and aminoacrylate in the β–active site; systems were subsequently neutralized by the addition of 13, 5 and 8 Na^+^ ions for the α/β–dimeric and isolated α– and β–monomeric units, respectively. Both LF and LB complexes have one Na^+^ ion placed close to the β–active site, as suggested by experiments. The LBR complex refers to a completely closed state of TRPS comprised of IGP and aminoacrylate in the β– and β–active sites of the complex, respectively. The carbonyl group of aminoacrylate was unprotonated, and six crystal waters were kept in the β–site. The Cs^+^ ion located close to the β–active site in the crystal structure (PDB entry 3CEP) was replaced with the Na^+^ ion; 13 more Na^+^ ions were added to neutralize the α/β–dimeric unit; and 5 and 8 Na^+^ ions were used to neutralize the isolated α– and β–monomers, respectively.

All 9 complexes were solvated by use of a 12 Å TIP3P water box with the *xleap* program in the Amber10 package [39]. Each dimeric unit has about 86000 atoms, whereas isolated monomers have ≤48000 atoms. The initial energy minimization for water molecules involved the *sander* program in Amber10. The NAMD 2.6 program was then used for further minimization, equilibration and production runs. Before equilibration, the systems were gradually heated from 250 to 300 K for 30 ps. The resulting trajectories were collected every 1 ps. The total trajectory lengths for the α/β–dimeric units were 60, 100 and 50 ns for LF, LB and LBR states, respectively. For the isolated α–monomeric units, the trajectories were 50 ns long for both the LF and LBR states, and 150 ns for the LB state. The production runs for the isolated β–monomeric units were 56, 126 and 45 ns for the LF, LB and LBR states, respectively. The NPT ensemble was applied, and periodic boundary conditions were used throughout the MD simulations. A temperature of 298 K was maintained by use of a Langevin thermostat with a damping constant of 2 ps^−1^, and the hybrid Nose-Hoover Langevin piston method was used to control pressure at 1 atm. The SHAKE algorithm was used to constrain the length of all bonds involving hydrogens; therefore, the time step was set to 2 fs. The non-bonded interactions were truncated at a distance of 14 Å with a switching beginning at 12 Å. The particle mesh Ewald method was used to treat long-range electrostatic interactions beyond the cut-off limit. The VMD program [48] was used for visualization and graphical representation. PyPAT script was used to analyze the H-bond network and MutInf [49] for the correlated motions in simulated trajectories. RMSF and entropy were calculated by Bio3D [50] and T-Analyst [51], respectively.

The Brownian dynamics simulation algorithm, together with a coarse-grained model (CGBD), was used to study the motions of the indole molecule in the channel formed by the α– and β–subunits. The CGBD simulation method has been well described [52]–[53]. In our simulation, the amino acids are represented by one bead placed at the Cα of each residue [54]. Most residues were assigned an effective radius from an existing publication [55]. For residues in the active sites and along the channel, the bead radius was measured by the distance between the Cα and side-chain based on a crystal structure (PDB entry 3CEP). For indole, each ring is represented by one bead, and an effective radius was based on the size of the pyrrole and benzene ring of 1.6 Å and 1.9 Å, respectively.

The protein is held rigid, and the motion of each bead of indole is simulated with use of the BD algorithm of Ermak and McCammon [56] and Shen et al. [57]. Although the slower protein fluctuations might have a role during indole channeling, the coupling between protein conformational changes and indole motion was not taken into account in this study [58]–[59]. Multiple protein conformations were chosen for the CGBD simulations. The diffusion coefficient used in the algorithm to move a bead was computed by the Stokes-Einstein equation, and the viscosity of water was set to 1 cp (T = 293 K). In our coarse-grained model, the beads of indole are linked by a virtual bond, and Coulombic and van der Waals interactions were applied for intermolecular interactions [54]–[56]. A Lennard-Jones type functional form was used for van der Waals interactions, U_vdw_ = 0.5[((r_i_+r_j_)/(r_ij_))^8^−1.5((r_i_+r_j_)/(r_ij_))^6^], where r_i_ and r_j_ are the effective radii of beads i and j, respectively. The Coulombic interaction was approximated by U_elec_ = q_i_q_j_/e_ij_ r_ij_, and a distance-dependent dielectric coefficient (e_ij_ = 4r_ij_) was used to avoid unrealistic *in vacuo* Coulombic interactions [60]–[61].

Conformations for the simulations are snapshots taken from 0, 6, 12, 24, 30, 40 and 50-ns MD simulations in the LBR state; 2, 12, 24, 48-ns MD simulations in the LF and LB states. All the snapshots were superimposed on the crystal structure (PDB entry 3CEP) and the coarse-grained indole molecule was placed in the same position in the α–active site shown in the crystal structure. For each snapshot, 500 different random number seeds were used to study motions of indole as it approached the β–active site. The simulations used a 50-fs time step and were run for 2–4 µs. A simulation was terminated if indole reached the β–site or escaped farther than 40 Å of the α–active site. If indole cannot reach the β–site within 4 µs, then we consider that the channel is blocked. If indole diffuses farther than 40 Å of the α–active site, then we consider that indole escapes, since it is unlikely that indole diffuses back to the active sites. We computed a distance between one bead of indole and the center of mass of the β–state in a given protein conformation to determine whether the indole reacted. If the distance was closer than 5 Å, then the indole reacted at the β–active site.

### Interaction energy and entropy calculation

The MM-PBSA approach was used to compute the ligand–protein interaction energies. The total energy E_tot_(**r**) can be divided into two terms: potential energy term, U(**r**), and solvation energy term, W(**r**), both functions of the coordinate **r**. The molecular mechanical energies were computed in a single MD step in the Sander module using a cutoff value of 40 Å for the non-bonded interactions. The solvation energy can be further decomposed into a Poisson-Boltzmann term, W_PB_, for electrostatic solvation free energy [62], and a cavity/surface area term, W_np_, for nonpolar solvation free energy [63]–[64]. For the electrostatic component of the solvation energy, the dielectric constant of the interior protein (solute) was set to 1, whereas an implicit solvent dielectric constant of 80 was defined for the solvent region. The nonpolar solvation free energy was approximated with the commonly used solvent-accessible surface area (SASA) model. The SASA was estimated with a 1.6-Å solvent-probe radius as implemented in Sander. Amber10 was used to compute all energy terms for each snapshot saved during the MD simulations, with waters removed [39]. The change in mean energy on molecular interactions can be split as follows:
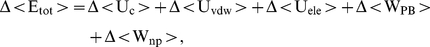
(1)where ΔU_c_ represents the changes in valance energy (bond, angle, dihedral and improper dihedral energies), ΔU_vdw_ represents van der Waals interactions, ΔU_ele_ represents Coulombic interactions, and ΔW_PB_ and ΔW_np_ represent polar and nonpolar solvation free energy, respectively. Each individual interaction energy term is calculated according to the following equations:
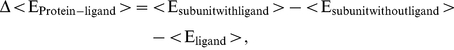
(2)where Δ<E_Protein-ligand_> is the ligand–protein interaction energy. Note that the valance energy term is cancelled during the calculations.

The configurational entropy S consists of conformational and vibrational parts, which describe the number and width of occupied energy wells, respectively [65]–[67], computed from each dihedral angle. The configurational entropy is calculated by the Gibbs entropy formula [68]:
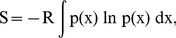
(3)where p(x) is the probability distribution of dihedral x and R is the gas constant. T-analyst was used to compute the Gibbs entropy, and only the internal dihedral degree of freedom of rotatable dihedrals is considered in the entropy calculations. The absolute temperature T was set to 298 K in this study. The change in configurational entropy of dihedrals of interest between a bound and free state can be obtained by TΔS_config._ = TS_bound_−TS_free_.

## Results/Discussion

TRPS is one of the best-characterized examples of an oligomeric enzyme with stringent allosteric regulation of the catalytic reaction. The enzyme has been proposed to cycle between a low-activity open conformation in the ligand–free (LF) state and a high-activity closed conformation in the ligand–bound–reference (LBR) state. The allosteric interactions are significantly influenced by the presence of α– and β–site ligands. Experiments suggest that destabilizing the α/β–interface or separating the α– and β–subunits loses allosteric communication, thus resulting in impaired catalysis, particularly at the α–site [22].

The 9 simulations starting from the α/β–dimeric unit or the isolated monomers with different states i.e. ligand–free (LF; IGP-free and/or aminoacrylate-free but PLP), ligand–bound (LB; IGP-bound and/or aminoacrylate-bound to the semi–open conformation proteins), and ligand–bound–reference (LBR; IGP-bound and/or aminoacrylate-bound to the closed conformation proteins) allow us to investigate the cooperativity between subunits and protein allostery induced by ligand binding. Moreover, we used Brownian dynamics simulations to study the coupling between the conformational changes and substrate channeling processes.

### Allosteric communications in the free and bound dimeric complex

**Figure 2:**
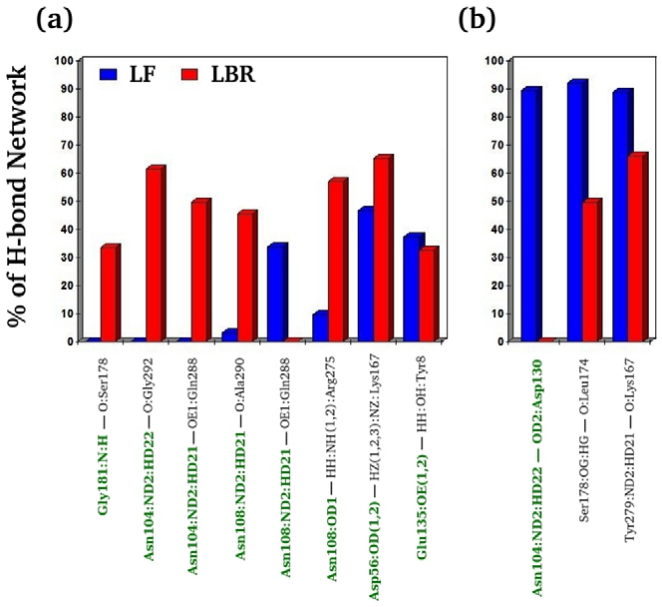
H–bond formations in different states. A quantitative comparison of H–bond formations in percentages for residues located at the α/β–interface of ligand–free (LF; blue bars) and ligand–bound–reference (LBR; red bars) dimeric units obtained from the molecular dynamics (MD) simulations of the TRPS complex. The formation of the H–bonds between α– and β–interfacial residues is given in (a), while (b) shows the formation of the H–bonds of the α– and β–interfacial residues within α– and β–subunits, respectively. The x–axis is labeled with the interacting pair of residues; the α–residues are labeled green and the β–residues are black.

Effective local or allosteric protein communication is a key to protein function. In most macromolecules, these communications are usually governed by non-bonded inter/intra-molecular interactions, such as van der Waals and electrostatic attractions and hydrophobic effects. Among these attraction forces, changes in hydrogen bond networks and surface areas are useful quantitative measurements for protein communication. Figure 1(b) demonstrated that interactions at the α/β–interface in TRPS combine hydrophobic interactions [69], and salt bridges and H-bonds. Experimental mutational studies for some of these interacting residues show that the salt bridges and H-bonds regulate allosteric and synergistic motions in the protein complex. A quantitative comparison of some H-bond networks, across the subunits and within the subunits, at the α/β–interface of LF and LBR dimeric units is shown in Figure 2(a, b). The analysis reveals a stronger communication at the α/β–interface of the LBR dimeric unit than at that of the LF dimeric unit. This finding suggests that binding of ligands in the α– and β–active sites of TRPS enhances the subunit communications, which are necessary to synchronize the catalysis taken in both α– and β–active sites located 25 Å apart from each other.

**Figure 3:**
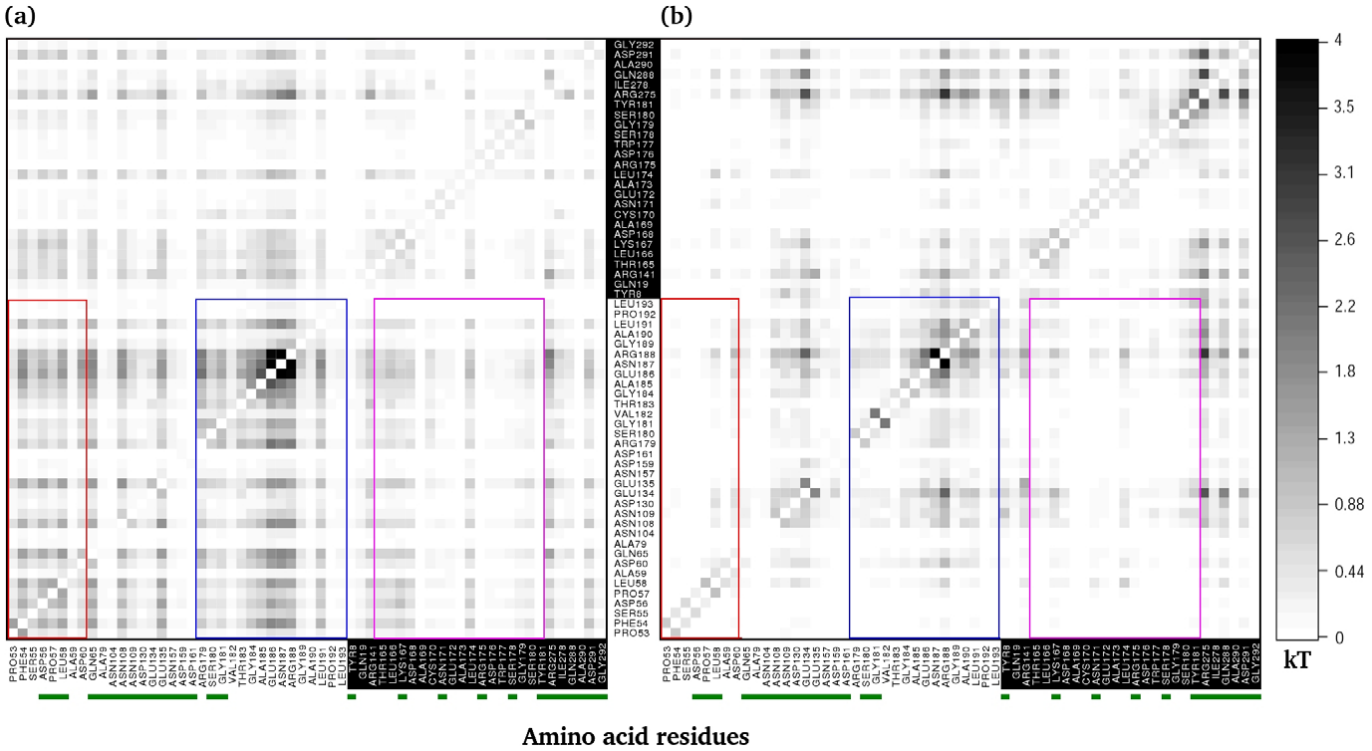
Comparison of correlated motions between different states. The comparison of correlated motions in the dimeric units with (a) ligand–free and (b) ligand–bound–reference states. The labeled α–residues on the x– and y–axes have a white background and the β–residues have a black background. Red, blue and pink rectangular boxes represent residues in α–L2, α–L6 and β–H6 of the COMM domain, respectively. The interfacial residues in both α– and β–subunits are underlined in green.

Correlated motions in proteins are ubiquitous and often related to protein functions. Assessing such correlations is therefore crucial for understanding protein function. Although we observed more inter-subunit interactions in the LBR state, the correlations are more pronounced in the LF state. The complex is also more flexible in the LF state, and the motions are not random but are in concert. Figure 3 shows a comparative correlation of regions important for subunit communication, such as α–L2, α–L6, β–H6 of COMM domain and residues at the α/β–interface of the TRPS dimeric unit in the LF and LBR states obtained by the use of the MutInf package [49]. With a few exceptions in the β–subunit, in general, the correlation is weaker at/near the dimeric interface in the LBR state than the LF state; loops α–L2 (red rectangular box) and α–L6 loops (blue rectangular box) show significant correlation in the LF state. The correlation map suggests that the α–subunit (α–L2, α–L6 and the interfacial residues) and β–H6 of the COMM domain (pink rectangular box) has weak correlation in the LBR state. A possible reason for a weaker correlation is that stronger inter-subunit interactions rigidify those regions (Tables 1 & 2) upon binding of the ligands, resulting in smaller magnitudes of correlative motions. We suggest that in the LF state, the concerted motions may help and guide the loops and the COMM domain to close when substrates bind to the active sites.

**Table 1:** Computed configuration entropy of important regions in the α–subunit from different states.

Configuration entropy from dihedral degree of freedom for the α–subunit (TS, kcal/mol)					
Protein system		α–L6	α–L2	α–active site	Total
α–subunit in the α/β–dimeric unit	LF	22.38	5.93	4.52	57.86
	LB	20.51	12.81	3.55	44.32
	LBR	8.64	−8.29	−1.18	0.21
Isolated α–monomer	LF	12.68	1.52	3.25	16.14
	LB	23.55	8.21	11.31	71.08
	LBR	7.17	3.80	2.15	14.58

Configuration entropy calculations (sum of Φ, Ψ and sidechain dihedral angles) of important α–subunit regions to the total effects of the whole α–subunit in ligand–free (LF), ligand–bound (LB) and ligand–bound–reference (LBR) dimeric and monomeric units.

**Table 2:** Computed configuration entropy of important regions in the β–subunit from different states.

Configuration entropy from dihedral degree of freedom for the β–subunit (TS, kcal/mol)				
Protein system		COMM Domain	β–active site	Total
β–subunit in the α/β–dimeric unit	LF	−4.08	3.02	−21.1
	LB	−2.69	−0.8	−35.43
	LBR	−16.29	−12.25	−53.58
Isolated β–monomer	LF	−2.65	−1.60	−40.11
	LB	−4.39	−2.36	−28.21
	LBR	−15.63	−8.67	−41.88

Configuration entropy calculations (sum of Φ, Ψ and sidechain dihedral angles) of important β–subunit regions to the total effects of the whole β–subunit in ligand–free (LF), ligand–bound (LB) and ligand–bound–reference (LBR) dimeric and monomeric units.

### Structural flexibility of isolated monomers versus α/β–dimeric unit

Fully closed protein conformations are believed to appear only when both α– and β–site ligands are present in the α/β–dimeric complex; they provide the optimized geometry necessary for enzyme catalysis. The closed conformations can optimize substrate–protein interactions to stabilize the substrate in the active site. To quantify the stability of substrates binding to the α/β–dimeric unit versus the isolated α– or β–monomer, we performed end-point energy calculation, also known as MM-PBSA calculations. Although more rigorous free energy calculation methods, such as umbrella sampling or metadynamics, need to be applied to get detailed free energy profile, it may need excessively large computational power to fully sample the energy landscape for a system with this big size [70]–[71]. A simple thermodynamic cycle and single-trajectory post-processing allow for efficiently computing the various contributions and differences in ligand binding to the dimeric and isolated monomeric units.

Because the catalytic rates are greatly reduced in the substrate-isolated monomeric complexes, we anticipated that both ligands might show weaker intermolecular attraction in the monomers. Unexpectedly, both α– and β–substrates in the substrate-isolated monomeric complexes showed fairly strong intermolecular attractions in the LBR TRPS state than in other states, which suggests that the monomers are nearly as stable as the dimeric unit. However, substrates in the LB monomer have higher inter-molecular energies and are unstable, and the conformations of ligand-monomer complexes deviate from their dimer conformations, especially in the α–subunit. Table 3 gives a comparison of ligand–protein interaction energies in the LB and LBR monomeric and dimeric complexes. The values of Δ*E_total_* for the isolated LBR monomers (α = −7.6 and β = −80.5 Kcal/mol) are similar to those in the LBR dimeric unit (α = −10.9 and β = −76.7 Kcal/mol), which suggests that the ligand–protein intermolecular attractions do not have significant differences between the isolated LBR monomers and the dimer. The changes in the electrostatic (Δ<*U_ele_+W_PB_*>) and the non–polar solvation (Δ*W_np_*) energy terms upon dissociation of the dimeric unit into the monomeric units are insignificant in the LBR states. In the LB states, the transition states during ligand binding processes, substrates interact weakly with the protein in both α– and β–monomers and the α/β–dimeric unit. Interestingly, the interactions are much weakened in the isolated α–monomer, which indicate that without forming an α/β–dimeric unit, ligand binding substantially disturbs the stability of the protein. Overall, both α– and β–substrates are less stable in the LB state than are ligands in the LBR state. The LB state is in association processes, whereas ligands are binding to TRPS. These findings suggest that the α/β–dimeric unit helps both α– and β–site ligands bind in the active sites and bring the proteins to the closed conformations through a systematically advanced allosteric communication across the α/β–interface. Absence of interface communication (i.e., the isolated monomers) detains the transition of open conformations to closed conformations and results in the deceleration of catalysis in monomer complexes.

**Table 3:** Calculated ligand–protein interaction energy for different states.

Ligand–Protein Interaction Energy in the α–subunit (Kcal/mol)							
Protein system		Δ*U_VDW_*	Δ*U_ele_*	Δ*W_PB_*	Δ*W_np_*	Δ*E_tot_*	Δ<*U_ele_+W_PB_*>
α–subunit in the α/β–dimeric unit	LB^a^	−33.6 (±3.5)	−66.6 (±13.5)	87.7 (±7.9)	18.8 (±1.4)	6.3 (±8.3)	21.3 (±8.9)
	LBR	−43.6 (±3.4)	−57.9 (±8.1)	71.1 (±5.1)	19.5 (±0.8)	−10.9 (±7.4)	13.1 (±8.6)
Isolated α–monomer	LB	−38.4 (±3.5)	−23.6 (±8.2)	57.4 (±9.7)	20.1 (±1.2)	15.4 (±8.2)	33.8 (±7.0)
	LBR	−39.2 (±3.7)	−70.2 (±7.3)	83.5 (±6.7)	18.2 (±1.1)	−7.6 (±8.3)	13.3 (±8.2)

Ligand–Protein Interaction Energy in the β–subunit (Kcal/mol)							
Protein system		Δ*U_VDW_*	Δ*U_ele_*	Δ*W_PB_*	Δ*W_np_*	Δ*E_tot_*	Δ<*U_ele_+W_PB_*>
β–subunit in the α/β–dimeric unit	LB^a^	−41.9 (±3.2)	−89.8 (±10.7)	134.3 (±10.4)	23.2 (±0.9)	25.8 (±9.2)	45.17 (±9.7)
	LBR	−33.9 (±6.1)	−181.0 (±31.2)	116.7 (±18.1)	21.4 (±1.1)	−76.7 (±14.7)	−64.3 (±16.7)
Isolated β–monomer	LB	−36.5 (±3.6)	−116.5 (±14.9)	153.4 (±10.7)	23.9 (±1.1)	24.1 (±9.5)	36.8 (±10.2)
	LBR	−36.7 (±5.1)	−178.1 (±21.5)	113.8 (±14.7)	20.5 (±0.9)	−80.5 (±11.6)	−64.2 (±11.9)

a Data from published paper [47].

Calculated ligand–protein interaction energy (kcal/mol) for ligand–bound (LB) and ligand–bound–reference (LBR) complexes of tryptophan synthase (TRPS) dimeric and monomeric units. The simulation length used for the energy calculations for the LBR monomeric and dimeric units is ∼50 ns, while the ∼60-ns length trajectories have been used for the LB states.

**Figure 4:**
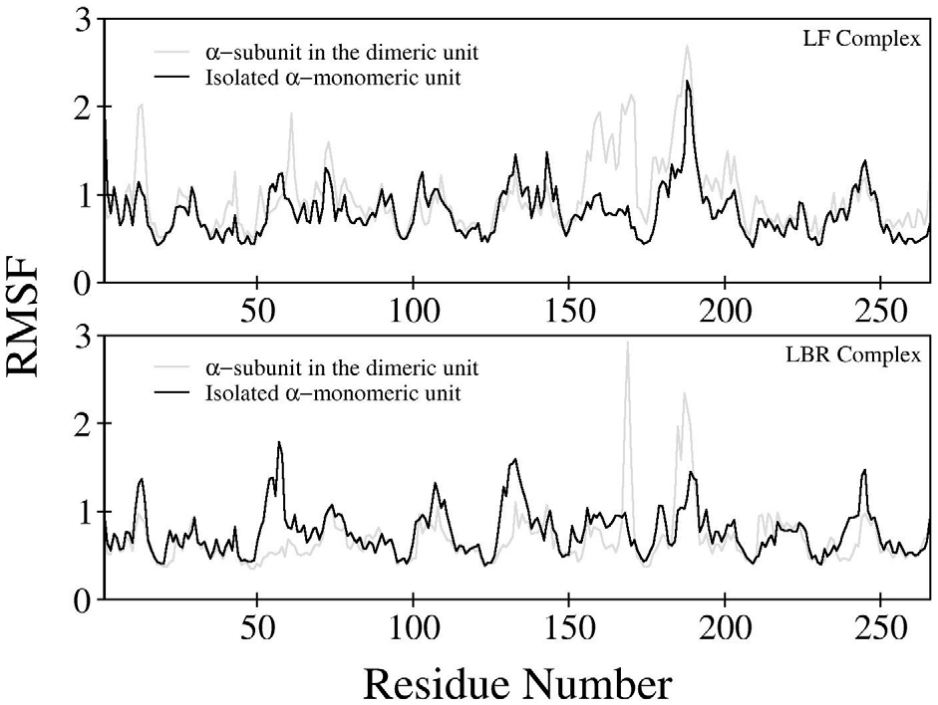
Plots of RMSFs. Comparative root mean square fluctuations (RMSFs) for the α–subunits in the dimeric complexes versus isolated α–monomeric units based on Cα atoms for each residue, averaged over the total simulation time of 50 ns of the ligand–free (LF) and ligand–bound–reference (LBR) states.

Of interest is knowing whether the isolated α– and β–monomeric units are more disordered than α/β–dimeric units, which may be less favorable for ligand binding. Therefore, we calculated the root mean square fluctuations (RMSFs) of Cα atoms and torsional entropy for each residue for the first ∼50 ns long trajectory of the LF and LBR states. Figure 4 shows a comparison of the RMSF values of the isolated α–monomeric unit and the α/β–dimeric units for both LF and LBR states, which match well with the trends of the fluctuations in the B-factors of the TRPS crystal structures. The RMSF plot clearly indicates that most of the regions in the isolated α–monomeric unit are more rigid, as compared to the α–subunit of the α/β–dimeric unit in the LF state, while an opposite trend can be observed for the LBR state. The effect of the ionic strength on the dynamics of the hydrophobic surface for the isolated α–monomeric unit in the LBR state seems negligible. The RMSF plot obtained from a 50 ns long explicit water MD simulation with 100 mM NaCl concentration is compared with those of the isolated α–monomeric unit and the α–subunit of the α/β–dimeric unit, and is given in Figure 1 in Text S1. For the β–monomeric units, in general, the difference in the RMSF values is insignificant. To quantitatively account for these flexibilities, torsional entropy was computed for the isolated monomeric and dimeric units in different states. The entropy computed for the peptide bond Ù angles was similar with all simulations, so we focused on other more flexible dihedral angles. The total entropic contributions for the backbone (Ø and Ö) and sidechains (SCs) indicated that in the LF state, both isolated α– and β–monomeric units are surprisingly more rigid than the dimeric unit (see Tables 1 & 2). Khare *et al.* [72] have observed a similar behavior in the wild-type Cu, Zn superoxide dismutase (SOD1) enzyme, where some residues are more rigid in the monomeric SOD1 as compared to dimer and are coherent with the NMR data. In TRPS, the difference is particularly significant in the sidechain rotation. Regions involved in ligand binding and closing the binding sites, such as α– and β–active sites, α–L6, α–L2, and β–COMM, show a pronounced decrease in sidechains motions of the isolated monomeric units, thus contributing to their rigidity. The hydrophobic binding interface between the subunits provides alternative contact points that allow sidechains of residues in the dimeric unit to adopt different binding conformations (data not shown). In addition, the correlated motions through non-direct sidechains contacts also increase protein flexibility, so such correlated motions vanish in the monomer.

Upon ligand binding, the protein flexibility was reduced largely in the dimeric unit; however, surprisingly, no significant entropic penalty was found in the isolated LBR monomers (TΔS = ∼1.6 and ∼1.7 kcal/mol for the α– and β–subunits, respectively). When the substrate binds in the α–active site, the dihedrals entropy of the α–subunit loses 57.6 kcal/mol in the dimeric unit (Table 1). Because ligand IGP has intra–molecular interactions and is not very flexible in its free state, the entropy loss from reducing the flexibility of a few rotatable bonds of IGP is not significant (∼2 kcal/mol). The difference is comparatively less sizable in the β–subunit; binding the β–ligand to the active site produces a protein dihedral entropy loss of 32.5 kcal/mol in the dimeric unit (Table 2). Interestingly and unexpectedly, without the partners, the isolated monomers are more rigid in the LF state. Although binding a chemical ligand to a protein may always result in losing the configuration entropy of the chemical compound, binding a protein ligand to a protein partner may have more complex behavior, such as gaining some flexibility in the TRPS system. A detailed study may be required to fully characterize and understand this behavior. As the LF monomer is more rigid, when ligand IGP binds to the active site, the flexibility changes between the LF monomer and the LBR monomer are also less substantial than those in the dimeric unit. In the LBR state, comparing the total entropy calculations shows that both α– and β–monomers are more flexible than the dimeric unit.

**Figure 5:**
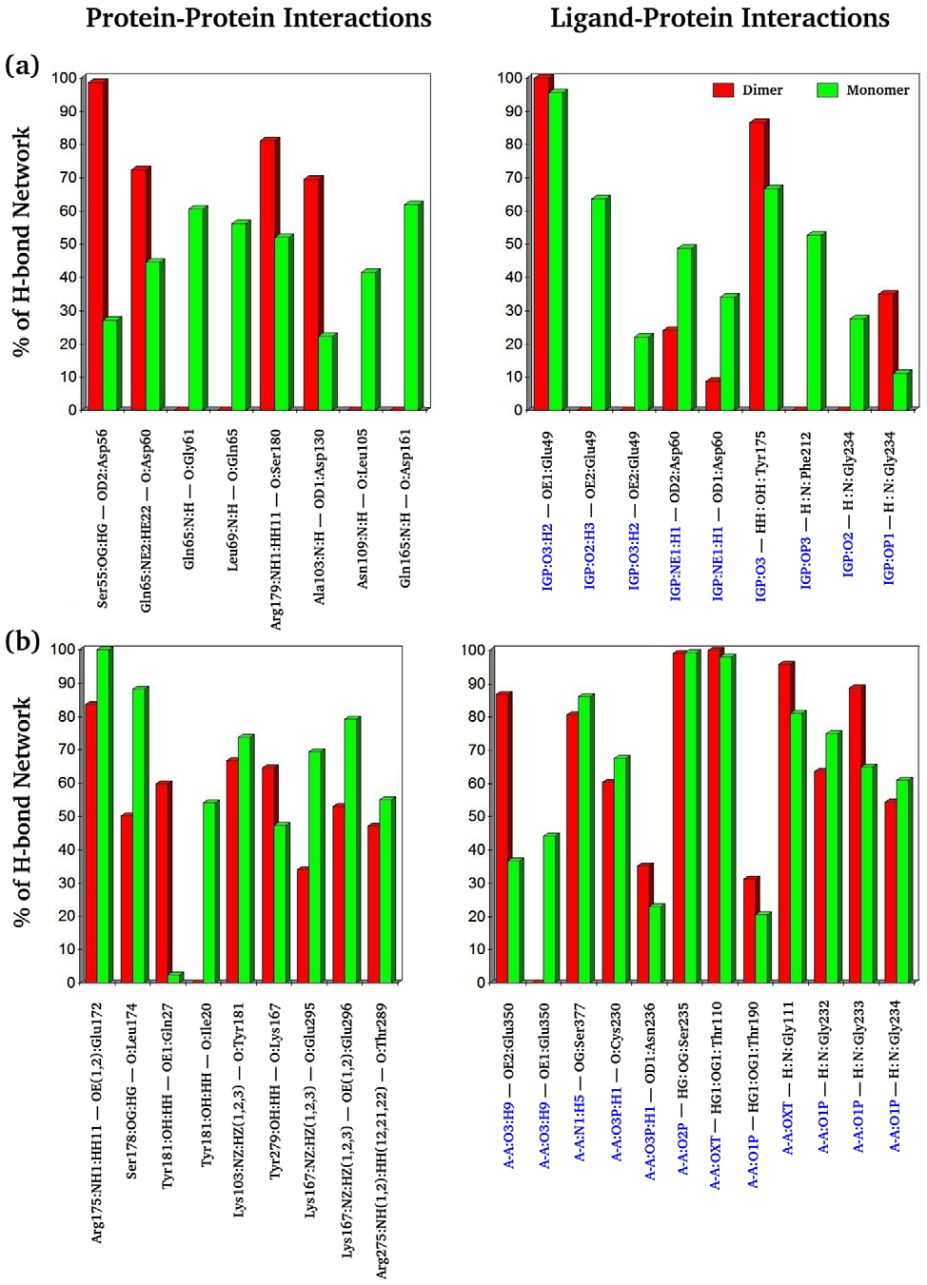
H–bond network in the dimeric and monomeric complexes. Quantitative comparison of the H–bond network for the dimeric and monomeric complexes of the ligand–bound–reference (LBR) state for residues located at the interface and residues interacting with ligands in the active sites. (a) α–subunit in the α/β–dimeric unit versus α–monomeric unit, (b) β–subunit in the α/β–dimeric unit versus β–monomeric unit. IGP and A-A are the α– and β–site ligands and represent 3-indole-D-glycerol-3′-phosphate and aminoacrylate, respectively. The x–axis is labeled with the interacting pair of residues, with the ligands blue and the protein residues in black.

**Figure 6:**
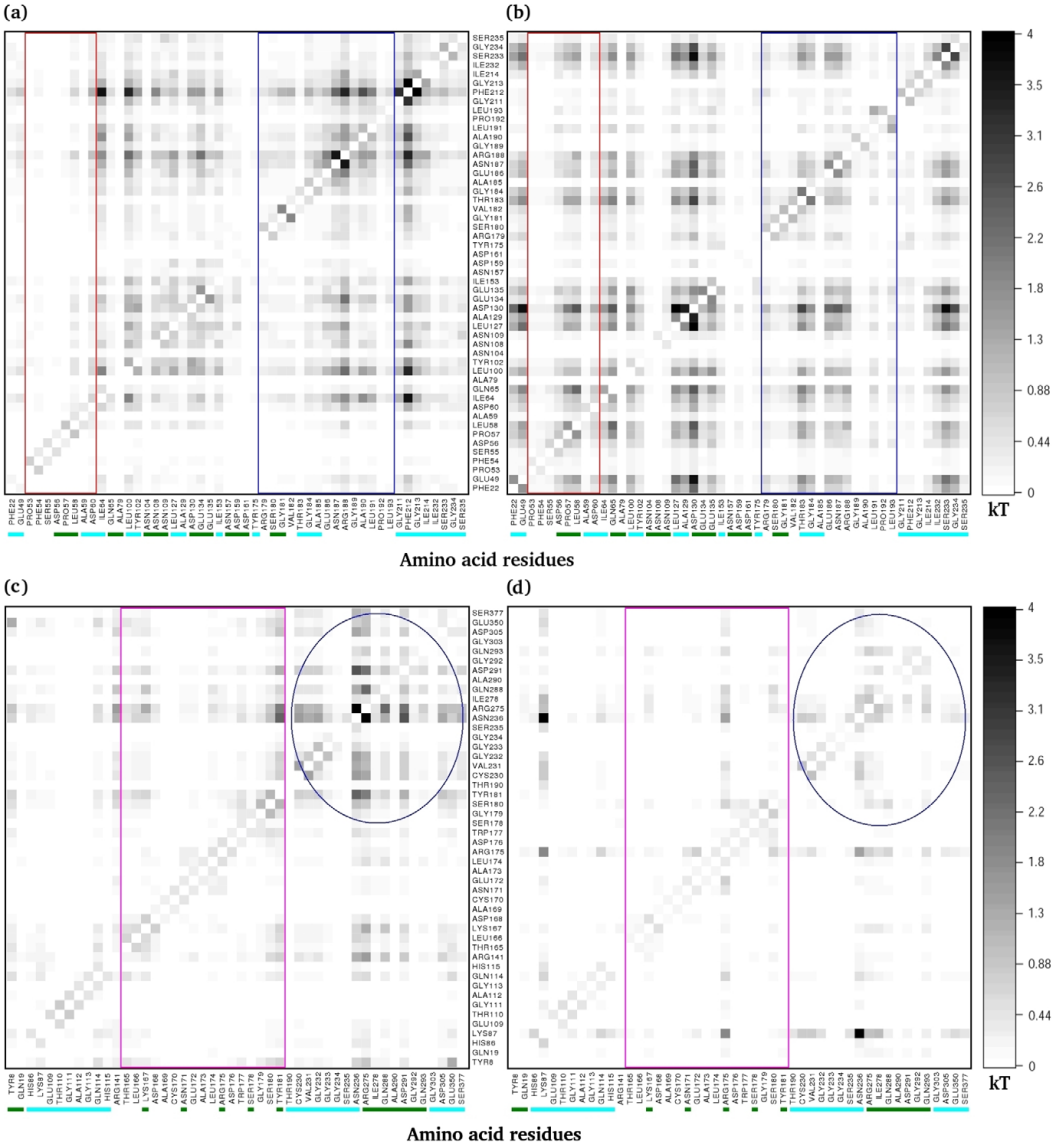
Comparison of correlated motions between the dimeric and monomeric complexes. Comparison of correlated motions between the α–subunit in the α/β–dimeric unit (a) versus the α–monomeric unit (b) and the β–subunit in the α/β–dimeric unit (c) versus the β–monomeric unit (d) for the ligand–bound–reference (LBR) state, calculated with MutInf. Red, blue and pink rectangular boxes represent residues in α–L2, α–L6 and β–H6 of COMM domain, respectively. The interfacial residues are underlined in green and the residues at the active sites are underlined in cyan.

In the isolated monomeric form, after ligand binding (the LBR state), the subunit has more freedom to change its conformation slightly to minimize the entropic penalty associated with gain of enthalpy in ligand–protein binding. For example, Glu49, Asp60, Gln65 and Asp130, which interact with the α–site ligand or communicate with the β–subunit, are able to form H–bonds with different atoms. The carboxylate or amide groups of these residues flip along with the sidechain (See Figure 2 in Text S1), which preserves the flexibility but forms multiple sets of H–bonds to gain reasonable substrate–protein interactions. Similarly, the LBR state shows a frequent flipping of carboxylate group coupled with the sidechain rotation in Glu350 and Glu172 of the β–active site in the isolated β–monomer. Figure 5 displays the percentage of H–bond networks for LBR monomeric and dimeric units for residues at the α/β–interface and active sites. Details regarding average distances and angles of H–bond are given in the Table 1 in Text S1. We found that in TRPS, generally, the loss of inter–subunit H–bond at the α/β–interface in the isolated monomeric unit is partly compensated by the formation of new H–bond networks within the subunit (see Figure 5a), as was also reported for other monomeric proteins [73]. We observed that the total number of H–bonds in the LBR monomeric states increased by at least 3–4 times as compared to the dimeric units (data not shown). Therefore, the isolated monomeric units are not less stable than the dimeric units energetically. In contrast, the dimeric unit has less room to adopt different protein conformations, which results in larger entropy loss in the LBR state. However, some residues (blue circles in Figure 6(c–d)) at the β–interface and in the β–active site show strong correlations with other interfacial residues and residues present in the β–active site of the LBR β–dimeric complex. These correlations are almost diminished in the isolated β–monomeric complex.

Overall, the effects of ligand binding and oligomerization on the 2 subunits are considerably different. The reasons may be that i) the β–subunit is larger and more rigid than the α–subunit, ii) the β–active site is buried within the subunit, right beneath the COMM domain and located relatively far from the interface as compared with the α–active site, and iii) the motion of the COMM domain in the β–active site is small as compared with the motion of loops in the α–subunit. The correlations within the β–subunit are minor as well (Figure 6). For example, residues involved in the communication with the α–subunit, 165 to 181 in the β–H6 of the COMM domain (pink rectangular box), are correlated weakly with the β–interface (green underline) and the β–active site residues (cyan underline) in both the monomeric and dimeric units.

### The power of two: Role of forming the dimeric complex

An increasing number of studies show that co-localization of proteins contributes to the efficiency of cellular signaling events and metabolic pathways [74]–[75]. TRPS is one of the model systems, and the dimeric unit is the minimal function structure. To mimic nature's synergy, one recent strategy is to engineer proteins that consider their spatial organization [76]. However, for enzymes such as TRPS, which are involved in regulation and synchronization in producing intermediate and final products, simply assembling multiple proteins in close proximity may not be enough. The dimeric unit forms a channel for efficient intermediate transportation, but the α– and β–subunits also use the inter–subunit interactions to assist in conformational transitions and synchronize the reactions in both active sites.

Our studies suggest that without a protein partner, both of the isolated α– and β–monomers form a stable and fully closed conformation when ligands are both bound in the active sites, which is the active form of the enzyme. However, the monomers, in particular the isolated α–monomer, may require an extended time to transit from an open/inactive form to a closed/active form. Forming the dimeric unit does not rigidify TRPS to form a pre–organized conformation for ligand access and to reduce entropic loss upon ligand binding. However, instead, it stabilizes the protein when the protein conformation is perturbed by the substrates during the binding processes. As a result, the dimeric unit has a smoother active–inactive transition. Notably, for both the isolated monomers and dimeric unit, the proteins sample both open and partially closed conformations, but the open (inactive) form is favored while the ligand is unbound in the LF state.

Presumably, because the hydrophobic interface provides alternative sidechain contacts and inter–subunit interactions, the dimeric unit is more flexible than the isolated monomer. Although the more flexible LF state in the dimeric TRPS results in larger configuration entropy loss upon ligand binding, we suggest that it also contributes to ligand recruitment. While a substrate is loosely bound to the binding site, the active–inactive transition rates increase, as was recently suggested by Zhou [77]. The binding sites are moving toward the fully closed conformation, and the binding mechanism gradually shifts from population shift (conformation selection) to induced fit [78]–[80]. However, as revealed by our simulations, the more unstable monomeric conformations in the ligand binding processes introduce a larger transition barrier; thus the transition rates can be decreased significantly. The dimeric unit uses the inter–subunit interactions to make the conformational transition easier.

In the LB state, the ΔE_tot_ of the isolated α–monomer is ∼9 kcal/mol larger than that of the α/β–dimeric unit, while the ΔE_tot_ for the β–monomeric and the dimeric unit lies within the standard error (see Table 3). The value suggests that the transition rate may be decreased by several orders of magnitude in the isolated α–monomer but reduced only a little in the isolated β–monomer. The results are in good agreement with experiments showing that the catalytic rate is ∼100 times slower in the isolated α–monomer but only 1.5 times slower in the isolated β–monomer as compared with the αββα tetramer [28]–[29]. The calculation further supports our conjecture that one major role of oligomerization in TRPS is to help the ligand binding processes.

In the LBR state, the isolated monomers show frequent flipping of the carboxylate group in key catalytical residues, such as α–Glu49, but the flipping rarely occurs in the dimeric unit. Multiple sets of H–bonds are established by the flipping of a carboxylate or an amide group and sidechain rotations, so the ligand–protein interactions are not weakened. However, the fluctuations can decrease the catalytic rate in the isolated monomeric units. Our work suggests that for residues directly involved in the catalysis, rigid sidechains are preferred for optimized protein function. A similar point has been concluded for the homomeric BX1 protein, whereby the protein has a rigid Glu134, the residue having the same role as α–Glu49, to enhance the catalytic rates [34].

### Coarse-grained Brownian dynamics simulation for intermediate channeling

**Figure 7:**
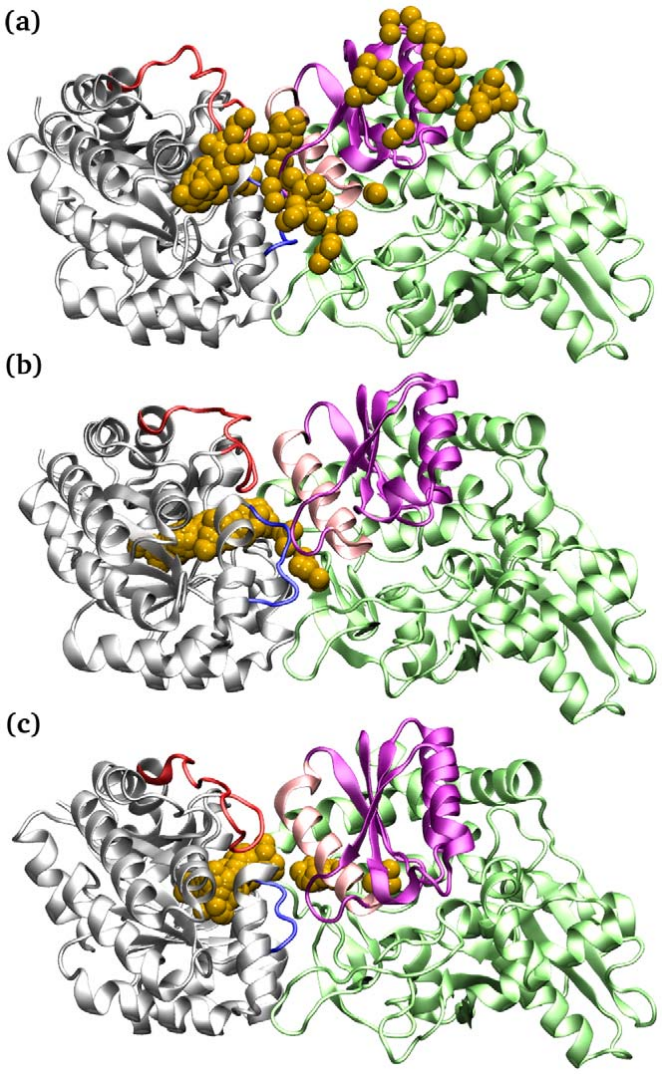
Brownian dynamics simulations in different states. Snapshots taken from the Brownian dynamics simulations of ligand–free (a), ligand–bound (b), and ligand–bound–reference (c) states of the α/β–dimeric units showing the leakage, blockage and passage of the indole intermediate, respectively, during the channeling process. Indole is represented by one yellow bead.

One of the unique features of the TRPS dimeric unit is substrate channeling. Conformational changes may affect the availability of the channel, and a fully closed conformation is necessary to avoid intermediate escape [81]. Protecting indole from diffusing away from TRPS is crucial for producing the final product, tryptophan, because the intermediate is relatively unstable in solution. Considering the significance of substrate channeling and the challenge of studying the process experimentally, we carried out CGBD simulation to explore the indole channeling processes (See Figure 7).

The transportation of indole in the LBR state is smooth and rapid. Almost all, 99.6%, of indole can reach the β–active site within 4 µs, and the average travel time is 39 ns. In contrast, on the basis of 4 different LF protein conformations taken from the atomistic simulations, only ∼50% of indole can reach the β–active site in the LF state. Note that we manually placed an indole to the α–active site in the LF state to simulate indole diffusion when TRPS is in open conformation. The travel time of indole towards the β–active site in the LF state is similar to that in the LBR state, but about a half of indole escapes the α–active site from the open α–loop6 (Figure 7a). In the LB state, where the protein is undergoing transition from an open to a fully closed form, indole does not always flow smoothly into the β–active site. A simulation from a snapshot taken from a 24-ns MD simulation showed that no indole could reach the β–active site, because of the channel blockage (Figure 7b). Other simulations from snapshots taken from 2- to 48-ns MD simulations revealed a leak in the α/β–interface resulting in only 73% of indole successfully arriving at the β–active site.

**Figure 8:**
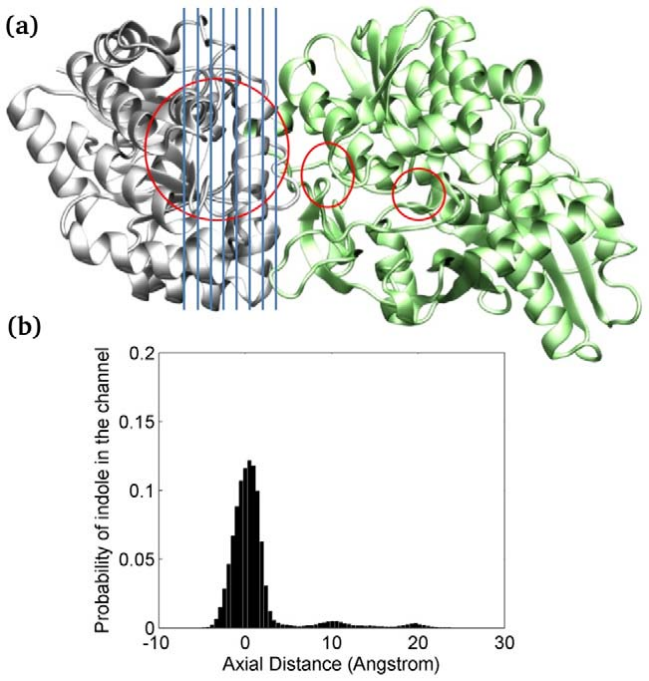
Analysis of indole distribution. Analysis of indole distribution in the channel from 360 individual coarse-grained Brownian dynamics (CGBD) simulations. The protein conformation is taken from a 30-ns MD simulation in the LBR state. (a) The channel is divided into 72 sections through the α–active site to the β–active site. Regions that indole spent most of the time during transportation are circled in red. The blue vertical lines shown in the plot does not reflect the real size of each section. (b) A histogram indicates regions of channel where indole preferentially resides.

Our work suggests that the channel also has a dynamic characteristic, which is substantially influenced by the conformational changes at the active sites. An efficient substrate channeling with a maximal success rate is only possible when both subunits are in fully closed conformation, which is in good agreement with the experiments. Since both indole and the channel are mainly non-polar, no major attraction forces that steer indole to diffuse from the α–site to the β–site were observed. Instead, indole spends longer time in positions that have larger cavities, as the molecule can freely diffuse to all direction before reaching the β–active site. The detailed channeling profile has been explored with the CGBD, and the population of indole staying in the channel formed by one conformation in the LBR state is shown in Figure 8b. The peaks correspond to large space appeared in the channel. Because our model provides fairly large space in the α–active site, indole usually needs to diffuse around the site before finding the right direction to move forward. Moreover, our trajectories show that indole may diffuse back and forth a couple of times in the channel before finally reaching the β–active site, which may be one reason that the diffusion time is an order of magnitude slower than indole diffusion in water. Our coarse-grained model keeps the protein rigid, so it cannot represent correlation between intermediate diffusion and protein conformational changes. However, as indole is a small and neutral molecule, it is unlikely to have prominent intermediate–protein correlations to accelerate the intermediate diffusion. For systems where protein motions strongly correlate with ligand channeling, a fully flexible protein system with the use of multi-bead coarse-grain models may need to be applied to more accurately capture the role of protein motions [82]–[83].

The significance of protein oligomerization in nature is widely recognized. TRPS is a good model system revealing the crucial role of oligomerization in assuring successful ligand binding and enhancing the rates of chemical catalysis. This study showed that the oligomerization of the α– and β–subunits not only provides a direct channel for efficient intermediate transportation but also permits allosteric cooperativity via inter–subunit communications to assist with conformational transitions necessary to synchronize the reactions in both α– and β–active sites.

## Supporting Information

Text S1 Supporting information for “The Role of Oligomerization and Cooperative Regulation in Protein Function: The Case of Tryptophan Synthase”
(3.10 MB DOC)
