# Supplementary material for: The Role of Oligomerization and Cooperative Regulation in Protein Function: The Case of Tryptophan Synthase
Source: PLoS Comput Biol. 2010 Nov 11;6(11):e1000994. doi: 10.1371/journal.pcbi.1000994 (PMC2978696; doi:10.1371/journal.pcbi.1000994)
Supplement: Text S1 — Supporting information for “The Role of Oligomerization and Cooperative Regulation in Protein Function: The Case of Tryptophan Synthase” (3.10 MB DOC) [file pcbi.1000994.s001.doc]

**The role of oligomerization and cooperative regulation in protein function: The case of tryptophan synthase**

**M. Qaiser Fatmi and Chia-en A. Chang***

Department of Chemistry, University of California, Riverside, Riverside, California 92521

E-mail: chiaenc@ucr.edu

Telephone: (951) 827-7263

Fax: (951) 827-4713.

**Definition of fold type II protein**

E.J. Goldsmith and coworkers (Protein Science, 1995, 4,1291-1304) have analyzed the similarities in amino acid sequences and secondary structures for several PLP-dependent enzymes and classified them into seven structural super-families. Fold type II proteins are typified by WSY (pdb entry 1WSY). The PLP−binding β−subunit of this enzyme contains two domains. In this alignment, E350 of WSY, which is involved in binding PLP, is largely conserved as E, D, or S.

**The details of docking parameters**

Autodock 4 and the graphical user interface AutoDockTools (1.5.1) were compiled for a Linux workstation. AutoDockTools 1.5.1 was used to establish the Autogrid points as well as visualization of docked protein-ligand structures. The protein target sites on both α and β subunits were specified according to the crystal structures. The grid centers were also established by centering the grid boxes on both active sites. The grid maps had a spacing of 0.375 and 0.243 Å for α and β subunits, respectively. The maximum number of energy evaluations per docking run was 2500000 and docking runs were varied as 20, 30 or 40 runs. All other docking parameters were left at the default values.

**Figure 1:** The comparison of the root mean square fluctuations of the isolated α−monomeric unit (high salt concentration; 100 mM NaCl concentration) with the isolated α−monomeric unit (0 mM salt conc.) and the α−subunit in the α/β−dimeric unit (0 mM salt conc.). The simulation length used for the root mean square fluctuations plots is ~50 ns.


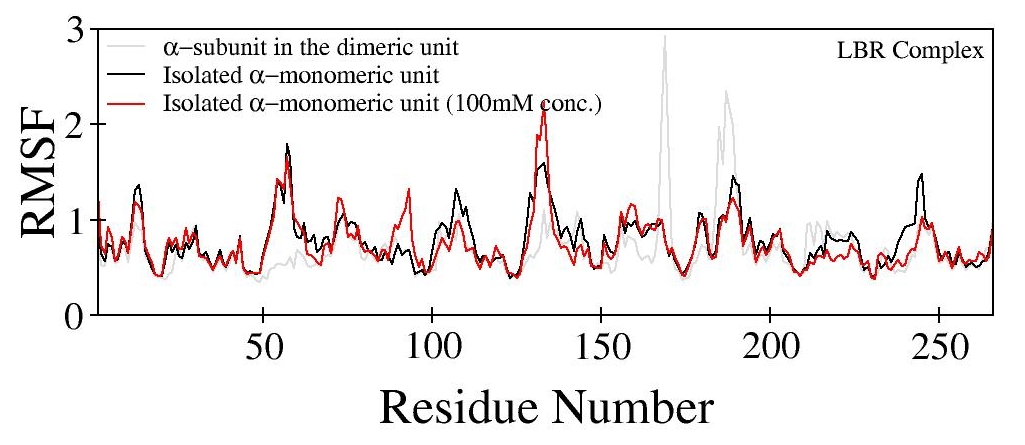


**Figure 2:** The comparison of the rotameric states of some acidic residues in ligand−bound−reference (LBR) dimeric and isolated monomeric units. The total simulation time is 30 ns, while each snapshot has been taken after 1 ps thus making a total of 3000 snapshots.

**(a)** α−**Glu 49**


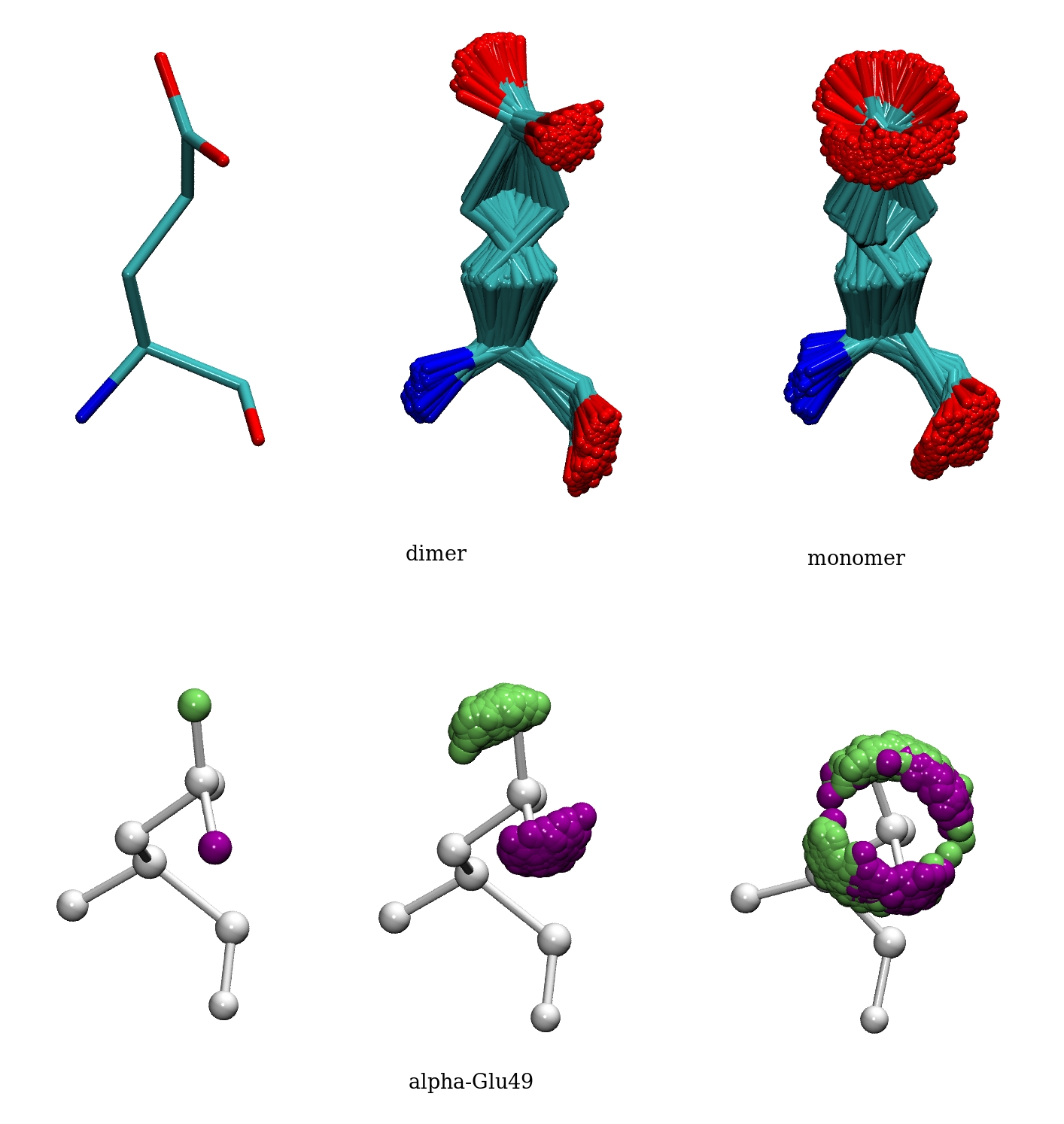


**Color code for top pictures**

Red = oxygen

Cyan = Carbon

Blue = Nitrogen

(True for other top pictures as well unless specified)

**Color code for bottom pictures**

Magenta and green color represents each oxygen atom of the carboxylate group. White color represents carbon backbone.

(True for other pictures as well unless specified)

**(b) α−Asp 130**

**
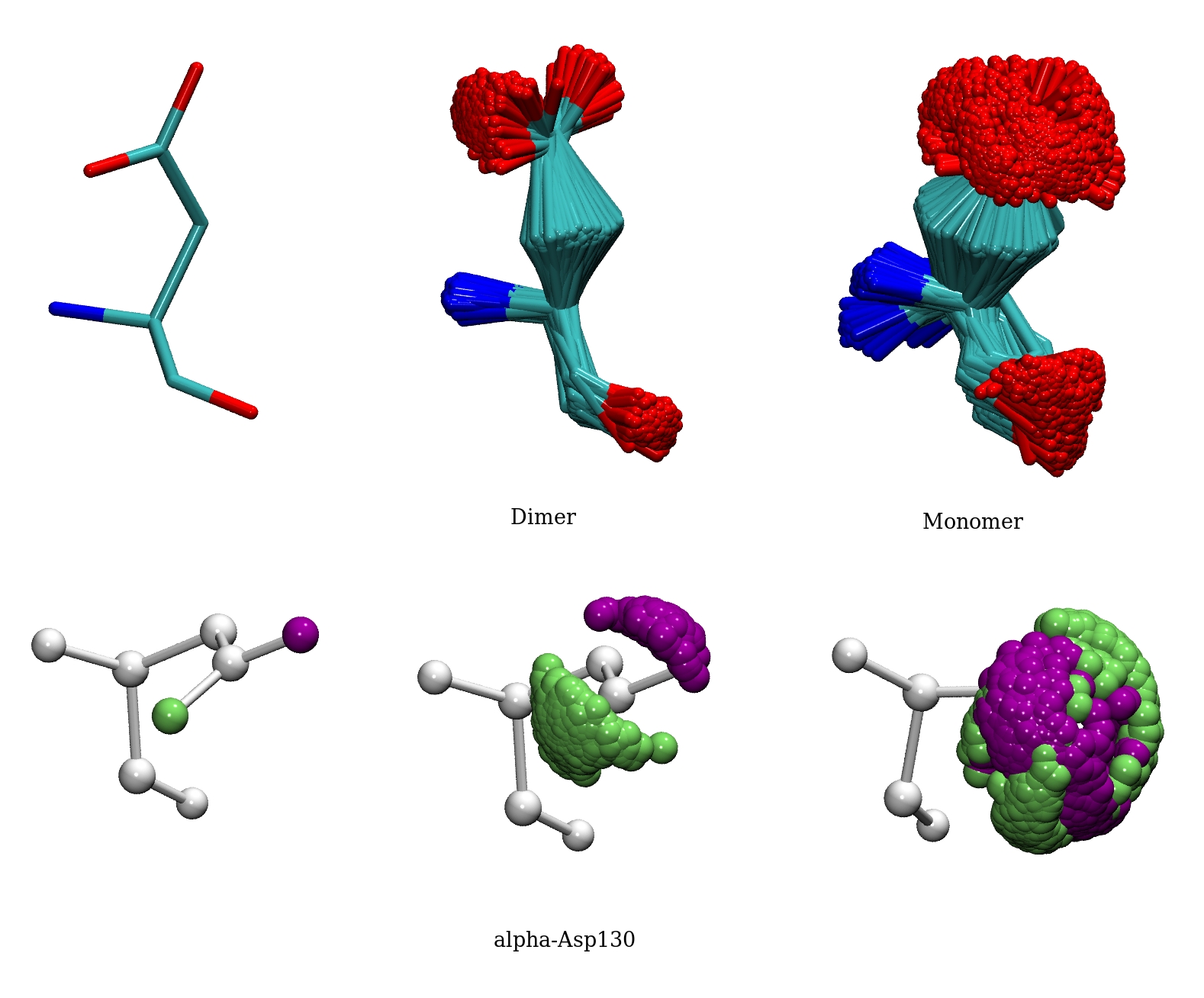
**

**(c) α−Asp 60**

**
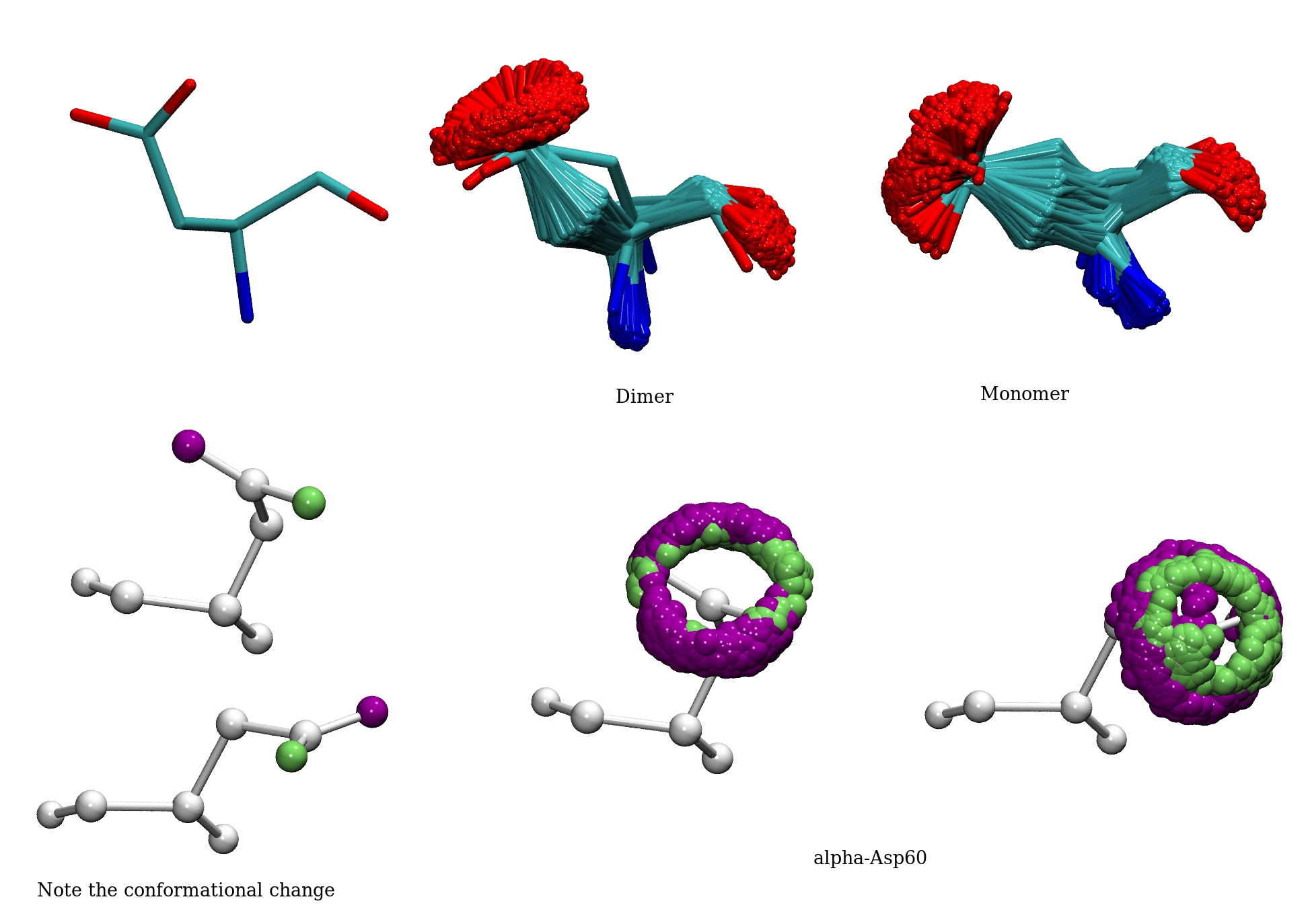
**

**(d) α−Gln 65**

**
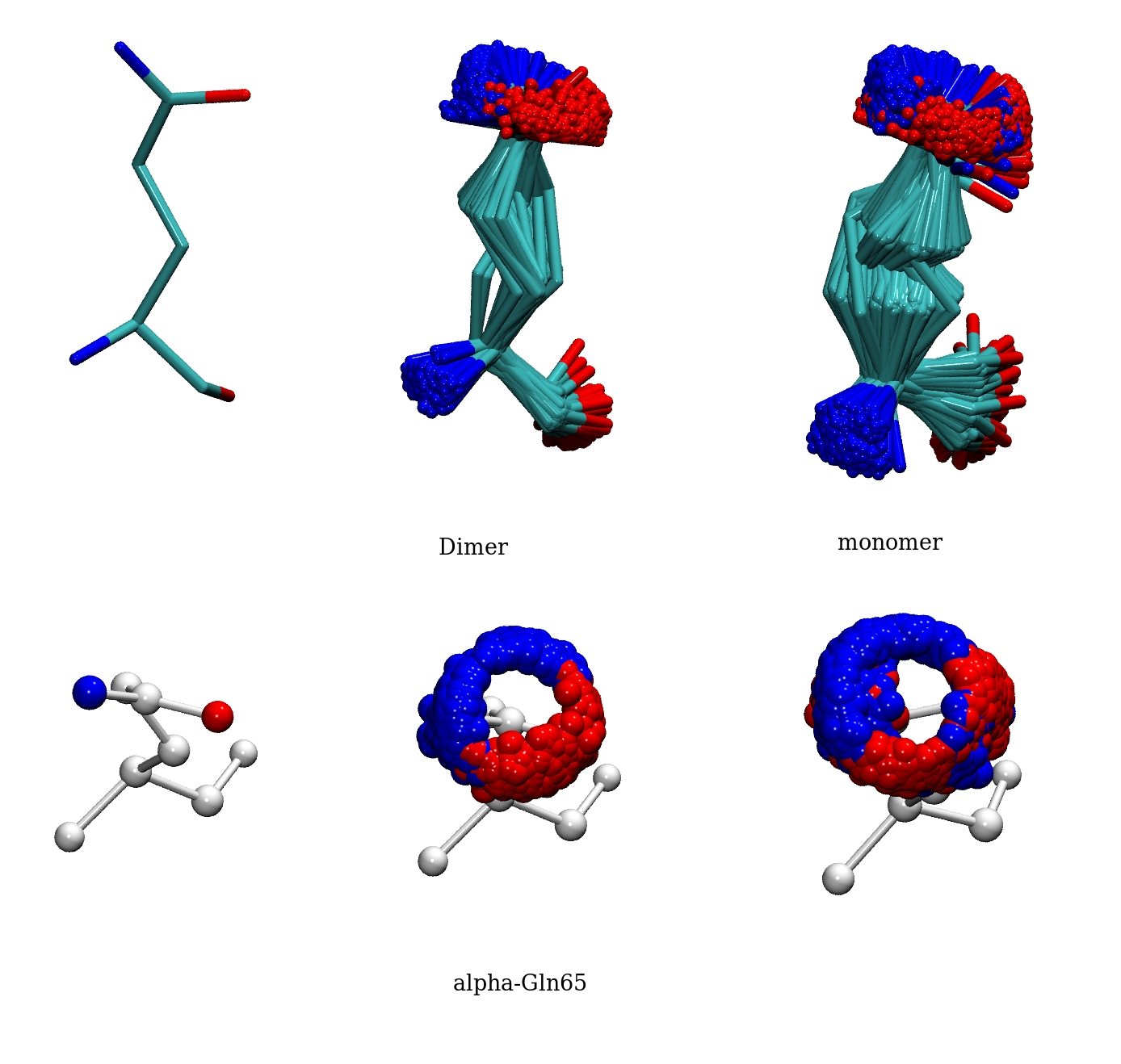
**

**Color code for top pictures**

Blue and red color represent nitrogen and oxygen atom, respectively, of the amide group

Cyan = carbon

**Color code for bottom pictures**

Blue and red color represent nitrogen and oxygen atom, respectively, of the amide group.

White = Carbon backbone

**(e) β−Glu 172**

**
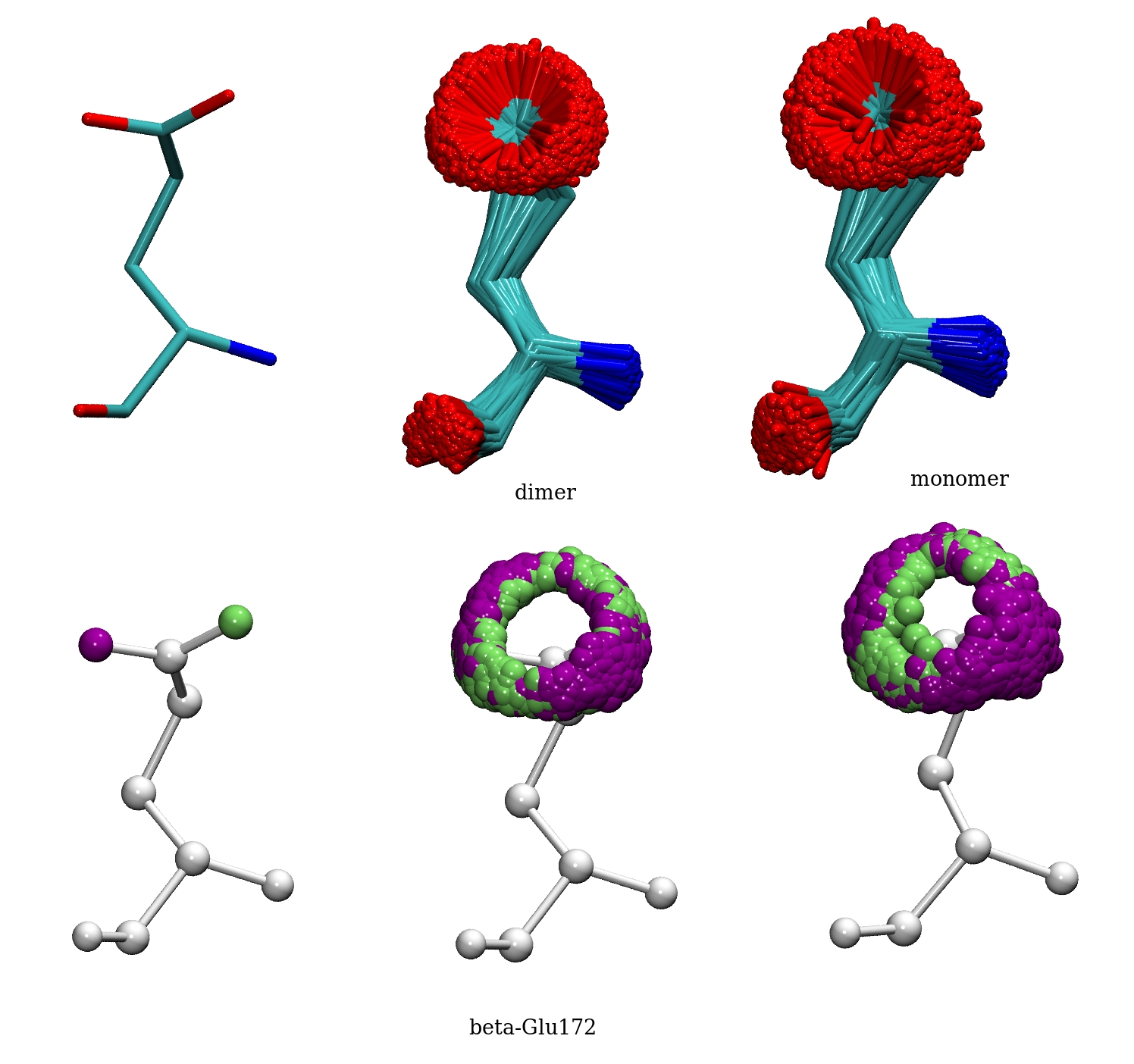
**

**(f) β−Glu 350**

**
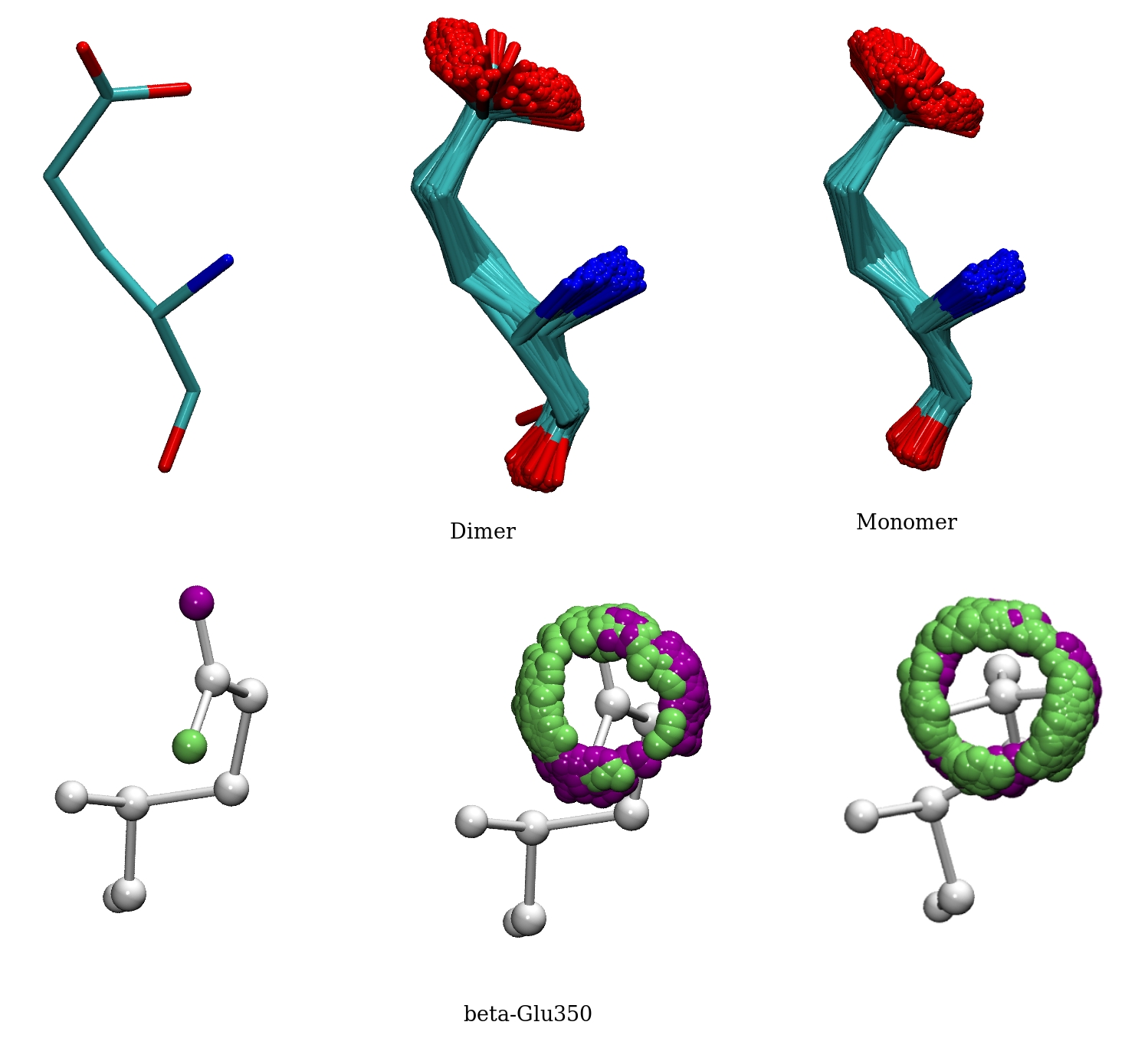
**

**Table 1:** A comparison of the hydrogen bond analysis for the ligand−bond−reference (LBR) dimeric and monomeric units.

**(a) H−bonding in the alpha−**active site

| **alpha−active site residues** | | **alpha−subunit in the Dimeric unit** | | | **alpha−monomeric unit** | | |
| --- | --- | --- | --- | --- | --- | --- | --- |
| **Accepter** | **Donor** | **%** | **Distance** | **Angle** | **%** | **Distance** | **Angle** |
| IGP:O3—H2 | OE1:Glu49 | 99.93 | 2.612(0.09) | 16.95( 8.24) | 95.53 | 2.654(0.12) | 18.40(10.62) |
| IGP:O2—H3 | OE2:Glu49 | - | - | - | 63.73 | 2.635(0.10) | 18.05(8.06) |
| IGP:O3—H2 | OE2:Glu49 | - | - | - | 22.07 | 2.842(0.12) | 40.39(12.91) |
| IGP:NE1—H1 | OD2:Asp60 | 24.13 | 2.861(0.09) | 26.42(11.07) | 48.8 | 2.860(0.09) | 26.44(12.13) |
| IGP:NE1—H1 | OD1:Asp60 | 8.73 | 2.863(0.08) | 30.15(13.10) | 34.2 | 2.878(0.08) | 30.08(14.38) |
| Tyr175:OH—HH | O3:IGP | 86.6 | 2.794(0.10) | 16.48(9.36) | 66.73 | 2.815(0.10) | 17.57(10.63) |
| Phe212:N—H | OP3:IGP | - | - | - | 52.8 | 2.866(0.08) | 32.75(11.36) |
| Gly234:N—H | O2:IGP | - | - | - | 27.48 | 2.899(0.07) | 43.50(8.59) |
| Gly234:N—H | OP1:IGP | 35.07 | 2.905(0.07) | 27.30(13.92) | 11.07 | 2.898(0.07) | 29.59(13.89) |

**(b) H−bonding within alpha−**subunit of alpha Interface residues

| **H−bonding within alpha subunit of alpha Interface residues** | | **alpha−subunit in the Dimeric unit** | | | **alpha−monomeric unit** | | |
| --- | --- | --- | --- | --- | --- | --- | --- |
| **Accepter** | **Donor** | **%** | **Distance** | **Angle** | **%** | **Distance** | **Angle** |
| ser55:OG—HG | **OD2:Asp56** | 98.67 | 2.657(0.10) | 14.29( 7.43) | *27.07 | 2.687(0.11) | 15.53( 8.37) |
| **Gln65:NE2—HE22** | O:Asp60 | 72.53 | 2.832(0.09) | 26.83(11.95) | 44.60 | 2.853(0.09) | 28.44(12.38) |
| **Gln65:N—H** | O:Gly61 | - | - | - | 60.53 | 2.878(0.08) | 17.33( 9.10) |
| Leu69:N—H | **O:Gln65** | - | - | - | 56.33 | 2.883(0.08) | 21.98(11.06) |
| Arg179:NH1—HH11 | **O:ser180** | 81.13 | 2.836(0.09) | 27.53(10.63) | 52.07 | 2.856(0.09) | 23.37(10.76) |
| Ala103:N—H | **OD1:Asp130** | 69.67 | 2.861(0.08) | 18.62( 8.70) | *22.40 | 2.834(0.09) | 18.90(10.40) |
| **Asn109:N—H** | O:Leu105 | - | - | - | 41.53 | 2.885(0.08) | 22.99(12.43) |
| Gln165:N—H | **O:Asp161** | - | - | - | 61.87 | 2.867(0.08) | 26.24(12.36) |

* sum of % for both OD1 and OD2 (multiple set of H−bonding)

Green color codes the residues at the alpha interface.

**(c) Beta active site**

| **Beta active site residues** | | **beta−subunit in the Dimeric unit** | | | **beta−monomeric unit** | | |
| --- | --- | --- | --- | --- | --- | --- | --- |
| **Accepter** | **Donor** | **%** | **Distance** | **Angle** | **%** | **Distance** | **Angle** |
| EAA:O3—H9 | OE2:Glu350 | 86.60 | 2.528(0.08) | 13.64( 6.48) | 36.60 | 2.528(0.08) | 13.60( 6.75) |
| EAA:O3—H9 | OE1:Glu350 | - | - | - | 44.07 | 2.524(0.07) | 13.65( 6.66) |
| EAA:N1—H5 | OG:ser377 | 80.47 | 2.840(0.08) | 20.31(11.48) | 86.13 | 2.839(0.08) | 20.65(11.76) |
| EAA:O3P—H1 | O:Cys230 | 60.20 | 2.754(0.11) | 18.89( 8.77) | 67.40 | 2.771(0.11) | 22.01( 9.58) |
| EAA:O3P—H1 | OD1:Asn236 | 35.20 | 2.706(0.11) | 17.11( 9.78) | 22.87 | 2.692(0.10) | 18.85(10.64) |
| ser235:OG—HG | O2P:EAA | 98.87 | 2.576(0.08) | 16.21( 8.79) | 99.13 | 2.591(0.09) | 15.16( 8.78) |
| Thr110:OG1—HG1 | OXT:EAA | 99.80 | 2.634(0.09) | 21.56(10.31) | 97.80 | 2.686(0.11) | 18.62( 9.71) |
| Thr190:OG1—HG1 | O1P:EAA | 31.27 | 2.732(0.11) | 16.57( 8.95) | 20.47 | 2.748(0.12) | 18.93( 9.16) |
| Thr190:OG1—HG1 | O2P:EAA | 21.87 | 2.783(0.12) | 40.82(11.67) | 13.60 | 2.798(0.12) | 37.32(13.67) |
| Lys87:NZ—HZ2 | O:EAA | 22.47 | 2.829(0.09) | 24.50(10.51) | 3.67 | 2.851(0.10) | 24.50( 9.14) |
| Lys87:NZ—HZ3 | O:EAA | 20.73 | 2.822(0.09) | 25.43(10.56) | 4.67 | 2.855(0.09) | 23.89(10.01) |
| Gly111:N—H | OXT:EAA | 95.73 | 2.779(0.08) | 18.01( 9.61) | 81.07 | 2.823(0.09) | 18.74( 8.99) |
| Gly232:N—H | O1P:EAA | 63.47 | 2.779(0.08) | 21.86( 9.51) | 74.93 | 2.785(0.09) | 24.52( 9.71) |
| Gly233:N—H | O1P:EAA | 88.60 | 2.831(0.08) | 21.30(11.29) | 64.93 | 2.851(0.08) | 33.45(13.11) |
| Gly234:N—H | O1P:EAA | 54.27 | 2.850(0.08) | 17.54( 9.37) | 61.00 | 2.863(0.08) | 19.48( 9.60) |

**(d) H−bonding within beta−**subunit of the beta Interface residues

| **H−bonding within beta−subunit of the beta Interface residues** | | **beta−subunit in the Dimeric unit** | | | **beta−monomeric unit** | | |
| --- | --- | --- | --- | --- | --- | --- | --- |
| **Accepter** | **Donor** | **%** | **Distance** | **Angle** | **%** | **Distance** | **Angle** |
| **Arg175:NH1—HH11** | OE(1,2):Glu172 | 83.60 | 2.804(0.09) | 20.92(9.49) | *103.99 | 2.821(0.09) | 23.90(11.46) |
| **ser178:OG—HG** | O:Leu174 | 50.13 | 2.782(0.11) | 17.38( 9.95) | 88.13 | 2.735(0.11) | 15.99( 8.98) |
| **Tyr181:OH—HH** | OE1:Gln27 | 59.73 | 2.753(0.12) | 20.87(11.02) | 2.40 | 2.710(0.12) | 24.97(11.87) |
| **Tyr181:OH—HH** | O:Ile20 | - | - | - | 54.13 | 2.741(0.11) | 23.89(12.64) |
| Lys103:NZ—HZ(1,2,3) | **O:Tyr181** | 66.87 | 2.816(0.09) | 31.36(14.43) | 73.80 | 2.810(0.09) | 29.98(14.25) |
| Tyr279:OH—HH | **O:Lys167** | 64.60 | 2.793(0.11) | 33.02(12.10) | 47.47 | 2.807(0.11) | 30.36(12.83) |
| **Lys167:NZ—HZ(1,2,3)** | O:Glu295 | 34.13 | 2.851(0.09) | 25.80(12.27) | 69.47 | 2.827(0.10) | 26.33(13.42) |
| **Lys167:NZ—HZ(1,2,3)** | OE(1,2):Glu296 | 53.01 | 2.778(0.09) | 28.38(13.45) | 79.2 | 2.767(0.09) | 24.81(12.97) |
| **Arg275:NH(1,2)--HH(12,21,22)** | O:Thr289 | 47.27 | 2.839(0.09) | 30.68(12.40) | 55.01 | 2.850(0.09) | 30.48(12.38) |

* highly dynamic: sum of all H-bond between NH(1,2)--HH(11,12,21,22) and OE(1,2)... forming multiple sets of H-bonding

Blue color codes for the residues at beta interface.

**(e) H−bonding at the alpha/beta−**subunit Interface

| **Residues at the alpha/beta−interface** | | **LBR Dimeric unit** | | |
| --- | --- | --- | --- | --- |
| **Accepter** | **Donor** | **%** | **Distance** | **Angle** |
| **Gly181:N—H** | **O:ser178** | 33.53 | 2.890(0.08) | 37.21(11.69) |
| **Lys167:NZ—HZ(1,2,3)** | **OD(1,2):Asp56** | 65.32 | 2.827(0.09) | 30.99(14.46) |
| **Asn104:ND2—HD22** | **O:Gly292** | 61.60 | 2.846(0.09) | 32.68(13.75) |
| **Asn104:ND2—HD21** | **OE1:Gln288** | 49.53 | 2.873(0.08) | 16.93( 9.25) |
| **Asn108:ND2—HD21** | **O:Ala290** | 45.60 | 2.872(0.08) | 22.24(11.35) |
| **Arg275:NH(1,2)—HH(12,21,22)** | **OD1:Asn108** | 56.87 | 2.837(0.08) | 29.41(11.9) |
| **Arg175:NH2—HH21** | **O:Pro57** | 83.13 | 2.831(0.09) | 25.91(10.81) |
| **Tyr8:OH—HH** | **OE(1,2):Glu135** | 32.67 | 2.753(0.12) | 22.27(11.12) |

Green color are alpha residues and Blue are beta residues
